# DNA barcoding ferns in an unexplored tropical montane cloud forest area of southeast Oaxaca, Mexico

**DOI:** 10.1038/s41598-021-02237-8

**Published:** 2021-11-24

**Authors:** Sonia Trujillo-Argueta, Rafael F. del Castillo, Daniel Tejero-Diez, Carlos Alberto Matias-Cervantes, Abril Velasco-Murguía

**Affiliations:** 1 Instituto Politécnico Nacional CIIDIR Oaxaca, Oaxaca, Mexico; 2 Tecnológico Nacional de México-Instituto Tecnológico de Oaxaca, Oaxaca, Mexico; 3 Carrera de Biología, Facultad de Estudios Superiores Iztacala, Universidad Nacional Autónoma de México, Mexico, Mexico; 4 CONACYT-Facultad de Medicina y Cirugía, Universidad Autónoma “Benito Juárez” de Oaxaca, Oaxaca, Mexico

**Keywords:** Biological techniques, Molecular biology, Plant sciences

## Abstract

DNA barcoding can be useful for species identification and phylogenetic analysis, but its effectivity has not been verified in most neotropical cloud forest plants. We tested three plastid barcodes, *rbcLa, matK*, and *trnH-psbA*, in selected pteridophytes, a well-represented group in these forests, from a little-explored area in Oaxaca, Mexico, applying the CBOL criteria for barcoding. We used BLASTn, genetic distance, and monophyly tree-based analyses employing neighbor-joining (NJ), maximum likelihood (ML), and Bayesian inference methods. Universal primers for *rbcLa* and *trnH-psbA* were successfully amplified and bi-directionally sequenced, but *matK* could not be amplified for most species. *rbcLa* showed the highest species discrimination in BLASTn (66.67%). *trnH-psbA* exhibited higher significant interspecific divergence values than *rbcL* and *rbcLa* + *trnH-psbA* (two-sample sign test, P value < 2.2e−16). Using NJ and ML phylogenetic trees, monophyletic species were successfully resolved (100%), differing only in support values and displaying full agreement with the most recent fern classification. ML trees showed the highest mean support value (80.95%). *trnH-psbA* was the only barcode that could detect the Elaphoglossoideae subfamily. Species discrimination did not increase using *rbcLa* + *trnH-psbA*. *rbcLa* is useful for fern barcoding, *trnH-psbA* is most helpful for phylogenetic analyses, and *matK* may not work as a universal barcoding marker*.*

## Introduction

Monitoring local biodiversity is fundamental for the development of conservation and sustainable strategies. This task requires a trustable species database, which is often lacking or incomplete for many regions on earth, particularly in the tropics, considered the richest in biodiversity^1^. Based on a recent estimation, there are about 8.7 million eukaryotic species on earth, of which more than 80% of plants remain to be described^2^. Classical morphological species identification often requires specimens in good conditions and reproductive structures, which are not always easy to obtain in field studies. Also, the higher phenotypic plasticity of plants makes it difficult to obtain an accurate identification, which frequently should be performed by a specialist of the taxon involved. DNA barcoding, a new molecular approach for species identification, overcomes these drawbacks. This technique requires only a piece of tissue of the specimen for species identification. DNA is extracted from a sample of tissue and amplified using universal primers. Then, the short fragment of amplified DNA is sequenced. The sequence is compared with those already published in a DNA database, such as the GeneBank at the National Center of Biotechnology Information (NCBI), or in the Barcode of Life Data system (BOLD) designed explicitly for DNA barcoding^3^. If the specimen has a DNA sequence that matches ≥ 99% of that already published in the database, then it is concluded that both sequences belong to the same species. Hebert et al. (2003) generated this technique^4^.

DNA barcoding using cytochrome oxidase I (*CO1*) has been successfully applied for animal species; but, in plants, *CO1* did not work, and more research is required. A *sine qua non* requirement for species identification using DNA barcoding is the existence of a published trustable sequence. The Consortium for the Barcode of Life's (CBOL) plant working group evaluated seven-candidate plastid DNA regions based on universality, sequence quality, and species discrimination. CBOL recommended using a core of a 2-locus combination of *rbcL* + *matK* as the plant barcode^5^. Other studies suggest using additional loci, including non-coding plastid regions, such as the intergenic spacer *trnH*–*psbA*^6–8^ and the nuclear marker *ITS*^9,10^. However, such universality has not been found in plants. Therefore, several authors propose a regional barcode for a wide range of ecological and conservation applications since the specimens are most likely to be identified using a restricted reference database^11,12^.

In Mexico, tropical montane cloud forests (TMCFs) account for 1% of the country's land area but have a higher plant and animal diversity concentration than any other Mexican ecosystem^13^. Pteridophytes are well represented in TMCFs in Southeast Mexico^14^. Oaxaca is the state where more diversity of pteridophytes has been observed^15^, but some places require increased sample collection^16^. Areas of difficult access, like the Mixteca highlands, had only one reported species^15^. To our knowledge, no new records were reported since this publication in this area. The purpose of this study is to evaluate the performance of three plastid barcodes: partial gene *rbcLa*, *mat*K, and the intergenic spacer *trnH-psbA*, using standard primers and under the three CBOL criteria (universality, quality, and species discrimination)^5^ to build a barcode library of pteridophytes in the Mixteca highlands, Oaxaca, Mexico.

## Material and methods

### Study site, determination, and vouchers

Twenty-nine samples of ferns from 11 families and two samples of lycopods were collected at a fragmented cloud forest at San Miguel Cuevas, Santiago Juxtlahuaca Municipality, Oaxaca state, México (17° 15′ 00.96″ N y 98° 02′ 57.34″), with 2187 m asl of mean altitude. Climate is temperate to semi-warm, and soils are rich in organic matter. Local authorities granted permission to visit their forests and to collect parts of the plants. Fresh plant vouchers were determined by Dr. Daniel Tejero Díez from UNAM FES Iztacala following Mickel and Smith^17^, recent taxonomic monographs^18^. Scientific names were checked in the Tropicos.org (https://www.tropicos.org/home) website and the Catalogue of Life (https://www.catalogueoflife.org/). The specimen names were compared with the type material in Jstor global plant (https://plants.jstor.org/). The herbarium vouchers were deposited at the National Mexican Herbarium, from Universidad Nacional Autónoma de Mexico (MEXU), and the herbarium from CIIDIR Oaxaca, Instituto Politécnico Nacional (OAX), pending for registration numbers due to the pandemic crisis.

All plant samples were collected in the field under permits issued by the municipal councils of San Miguel Cuevas, Juxtlahuaca. In no case was the full plant collected; the process of collecting the samples did not kill the plants, which were left alive in their original places. None of the samples collected belong to the near-threatened, vulnerable, endangered, critically endangered, extinct in the wild, or extinct of the IUCN red list (accessed September 7, 2021). Of all species studied, only three belong to the category “least concern” according to the IUCN: *Asplenium monanthes*, *Cystopteris fragilis*, and *Pteridium feei*. The rest of the species were not registered in the IUCN red list, probably because of insufficient data. We, therefore, encourage more studies to assess the IUCN status of these plants.

### Isolation, amplification, and sequencing of DNA

The number of individuals sampled per taxon was generally one and less frequent 2. Several leaves of each botanical sample were placed in a Ziplock^®^ bag and kept at − 20 °C in a freezer until processed. Genomic DNA was extracted from 2 mg leaf tissue with FastDNA SPIN kit and FastPrep^®^ (MP Biomedicals, USA) equipment. DNA concentration (ng/µl) and purity (260/280A) from total DNA extracted were measured in a Biophotometer (Eppendorf^®^). Three chloroplast DNA regions were used for amplification: *rbcLa*, *matK*, and the intergenic spacer *trnH*-*psbA*. We used standard primers from the Canadian Center for DNA Barcoding (CCDB)^19^ and a second set of primers for MatK^20^ (see Table 1 for primer DNA sequences). All three chloroplast regions were amplified using a 25 μl volume of reaction mixture: 5 µl MyTaq Buffer reaction (kit MyTaqDNA Polymerase Bioline), 1 μl of forward primer, 1 μl of reverse primer, 0.2 μl of MyTaq Polymerase, 15.8 μl of nuclease-free water, and 2 μl of isolated genomic DNA template. PCR reaction was carried out in an Applied Biosystems Veriti^®^ thermocycler. PCR temperature cycling programs followed Fasekas et al. protocols^24^. PCR for *rbcLa*: 94 °C for 4 min; 35 cycles of 94 °C for 30 s, 55 °C for 30 s, 72 °C for 1 min; final extension 72 °C for 10 min. PCR for *matK* 94 °C for 1 min; 35 cycles of 94 °C for 30 s, 52 °C for 20 s, 72 °C for 50 s; final extension 72 °C during 5 min. PCR for *trnH-psbA* (for ferns and allies) 94 °C for 4 min; 2 cycles of 94 °C for 45 s, 50 °C for 45 s, 72 °C for 1 min; 35 cycles of 94 °C for 45 s, 45 °C for 45 s, 72 °C for 1 min; final extension 72 °C for 10 min. Amplified PCR products were detected using agarose gel electrophoresis (1.2% agarose gel TBE) under UV light by staining with GelRed Nucleic Acid (Biotium). PCR products were purified using the EZ-10 Spin Column PCR Products Purification Kit (Biobasic). All PCR products were sequenced by Capillary Electrophoresis Sequencing (CES) in an ABI 3730xl System at the Macrogen sequencing facility (Macrogen Inc., Seoul, Korea).

**Table 1:** Primer sequences used for DNA amplification of chloroplast regions *rbcLa*, *matK,* and *trnH-psbA.*

Chloroplast DNA región /primer name	Primer sequence 5´- 3´	References
***rbcLa***		
rbcLa-F	ATGTCACCACAAACAGAGACTAAAGC	^21^
rbcLa-R	GTAAAATCAAGTCCACCRCG	^6^
***matK***		
MatK-1RKIM-f	ACCCAGTCCATCTGGAAATCTTGGTTC	Ki-Joong Kim, pers. comm
MatK-3FKIM-r	CGTACAGTACTTTTGTGTTTACGAG	Ki-Joong Kim, pers. comm
FERmatK fEDR	ATTCATTCRATRTTTTTATTTHTGGARGAYAGATT	^20^
FERmatK rAGK	CGTRTTGTACTYYTRTGTTTRCVAGC	^20^
***trnH-psbA***		
trnHf_05	CGCGCATGGTGGATTCACAATCC	^22^
psbA3_f	GTTATGCATGAACGTAATGCTC	^23^

### DNA alignment

*rbcLa* and trnH-psbA sequence chromatograms were manually edited and assembled into contigs using CodonCode Aligner v.9.0.1 http://www.codoncode.com/aligner/. Due to the low amplification frequency, *matK* was excluded from further evaluations. Consensus sequences were generated and aligned using MUSCLE^25^. These alignments were examined by eye and corrected when necessary.

### BOLD and Genebank

The project was registered under the name “Ferns and allies of a humid temperate forest in Oaxaca, México” project code FERNO (http://www.boldsystems.org) at The Barcode of Life Data System (BOLD), which is an informatics workbench devoted to the acquisition, storage, analysis, and publication of DNA barcode records^3^. Three files were submitted to BOLD. First, the Specimen data file included detailed voucher information, scientific names of taxa sampled, collection dates, geographical coordinates, elevation, collectors, identifiers, and habitat. Then, an image file was submitted with high-quality specimen images from each fern and lycopod collected. Finally, a trace file was submitted along with primers, the direction of sequences, and the molecular marker. Sequences uploaded to BOLD were edited and aligned in FASTA format and referenced by Sample IDs. Sequences were also submitted to GeneBank.

### Species discrimination

To evaluate species discrimination in *rbcL* and *trnH-psbA* sequences, we used three approaches: The Basic Local Alignment Search Tool for nucleotide (BLASTN) method^26^, which searches against the sequence database available online by the National Center for Biotechnology Information (NCBI) https://www.ncbi.nlm.nih.gov, genetic distance and monophyly tree-based analyses using Neighbor-Joining (NJ), Maximum Likelihood (ML) and Bayesian Inference (BI) analysis.

Following previous studies^10,27^, query sequences having ≥ 99.0% identical sites to sequences in the database were taken as correct assignments. Percentage species resolution was calculated for each plastid region. The combined *rbcL* + *trnH-psbA* species resolution was calculated as the cumulative percentage of each molecular marker^28^.

To determine the best fit model of nucleotide substitution for phylogenetic analyses jModel test v.2.0.^29^ was used. We found the general time-reversible model plus gamma distribution (GTR + G) as the best fit for *rbcLa*, which states for variable base frequencies with symmetrical substitution rates. For *trnH-psbA*, the best fit was achieved with the transversion plus gamma distribution model (TVM + G), with variable base frequencies, equal variable transversion rates, and transition rates. The data set of each plastid region was analyzed alone and in combination. Sequences of *rbcL* and *trnH-psbA* were concatenated into a single matrix *rbcL* + *trnH-psbA* with Mesquite^30^.

Genetic distance and NJ bootstrap consensus tree were inferred from 1000 replicates, and the evolutionary distances were computed using the Kimura 2-parameter method with gaps/missing data treatment adjusted using pairwise deletion. Genetic distance and neighbor-joining trees were constructed in MEGAX^31^ for each plastid barcode alone and in combination. To evaluate which plastid barcode showed more interspecific divergence and checked for any improvement using these barcodes in combination, we conducted two-sample sign tests with the BSDA package in R^32^.

We ran ML analyses with the IQ-TREE web server (http://iqtree.cibiv.univie.ac.at). Internal node support, bootstrap analyses were calculated using 1000 iterations. Tree inference using Bayesian analysis was run on MrBayes 3.2.2 on XSEDE via the CIPRES supercomputer cluster (www.phylo.org) for 10 million generations. The tree-based methods (NJ, ML, and BI) evaluated which tree produced the greatest species resolution and whether the barcode sequences form monophyletic groups.

## Results

### Studied species

Table 2 shows the fern and lycopod species determination and that were used for the barcoding analysis.

**Table 2:** Ferns and lycopods collected at Mixteca Alta, Oaxaca, samples’ ID, and species determination.

Sample ID	Fern family	Species
AVM4	Pteridaceae	*Jamesonia flexuosa* (Humb. & Bonpl.) Christenh
AVM42	Marattiaceae	*Marattia weinmanifolia* Liebm
AVM57	Cyatheaceae	*Cyathea bicrenata* Liebm
AVM68	Athyriaceae	*Diplazium lonchophyllum* Kunze
AVM77	Dryopteridaceae	*Dryopteris wallichiana* (Spreng.) Hyl
AVM127	Blechnaceae	*Blechnum appendiculatum* Willd
AVM128	Dryopteridaceae	*Phanerophlebia macrosora* (Baker) Underw
AVM130	Dryopteridaceae	*Dryopteris wallichiana* (Spreng.) Hyl
AVM132	Aspleniaceae	*Asplenium monanthes* L
AVM154	Dicksoniaceae	*Lophosoria quadripinnata* (J.F. Gmel.) C. Chr
AVM155	Marattiaceae	*Marattia weinmanifolia* Liebm
AVM157	Cyatheaceae	*Cyathea fulva* (M. Martens & Galeotti) Fée
AVM171s	Dryopteridaceae	*Arachniodes denticulata* (Sw.) Ching
AVM224	Cystopteridaceae	*Cystopteris fragilis* (L.) Bernh
AVM228	Polypodiaceae	*Polypodium conterminans* Liebm
AVM247	Dennstaedtiaceae	*Pteridium feei* (W. Schaffn. ex Fée) Faull
AVM267g	Pteridaceae	*Gaga hirsuta* (Link) Fay W. Li & Windham
AVM268	Polypodiaceae	*Polypodium subpetiolatum* Hook
AVM277	Pteridaceae	*Pteris muricella* Fée
AVM279	Dryopteridaceae	*Polystichum fournieri* A.R. Sm
AVM280	Aspleniaceae	*Asplenium monanthes* L
AVM288	Dryopteridaceae	*Elaphoglossum xanthopodum* Mickel
AVM303	Pteridaceae	*Adiantum andicola* Liebm
AVM304d	Dryopteridaceae	*Elaphoglossum petiolatum* (Sw.) Urb
AVM306	Dryopteridaceae	*Elaphoglossum xanthopodum* Mickel
AVM319	Dryopteridaceae	*Polystichum fournieri* A.R. Sm
AVM403	Blechnaceae	*Blechnum appendiculatum* Willd
AVM404	Cystopteridaceae	*Cystopteris fragilis* (L.) Bernh
	**Lycopod family**	
AVM30-01	Lycopodiaceae	*Lycopodium clavatum* L
AVM31	Lycopodiaceae	*Diphasiastrum thyoides* (Humb. & Bonpl. Ex Willd.) Holub

### PCR amplification and sequencing success

Using universal primers from CCDB of *rbcL* and *trnH-psbA,* fern DNA was successfully amplified in most cases (96.77%). Nevertheless, we could not get *matK* amplifications (Table 3). Furthermore, a second set of primers for *matK* designed specifically for most ferns^20^ were tested, and we could only get 19.36% amplification. In particular, we could only get amplicons from: *Phanerophlebia macrosora, Dryopteris wallichiana, Asplenium monanthes, Lophosoria quadripinnata, Cystopreris fragilis,* and *Blechnum appendiculatum.* Therefore, further evaluations only include *rbcL* and *trnH-psbA*.

**Table 3:** Proportion of samples successfully amplified and sequenced from three barcoding plasmid regions using tissues from different species of ferns and lycopods of the Mixteca Alta, Oaxaca, Mexico.

Plastid DNA region	No. individuals sampled	Amplification success (%)	Bidirectional Sequences obtained F & R (%)	Bidirectional high quality sequences > 250 bp (%)
*rbcLa*	31	96.77 (30/31)	100 (60/60)	93.33 (56/60)
*matK* universal primers^19^	31	0.00	N/A	N/A
*matK* second set primers^20^	31	19.36 (6/31)	N/A	N/A
*trnH-psbA*	31	96.77(30/31)	100 (60/60)	80.00 (48/60)

The sequencing success rate (bidirectional high-quality sequences > 250 bp) was higher for *rbcL* (93.33%) than for *trnH-psbA* (80.00%) (Table 3).

### Blast discrimination, BOLD, and GeneBank

We found 100% resolution per family and genera of ferns and lycopods using BLASTn in both plasmid barcodes. We contributed to new species in the GeneBank Taxonomy Database for DNA sequences for *rbcLa* (8 species), and *trnH-psbA* (16 species). With the accessions already published, we found that *rbcLa* could discriminate to species level 66.67% of the cases, whereas *trnH-psbA* discriminates 50%, and *rbcLa* + *trnH-psbA* 60.61%. The best BLAST match identification per species for *rbcLa* plastid barcode is shown in Table 4 and for *trnH-psbA*, in Table 5.

**Table 4:** BLAST search best match found on GeneBank for ferns and allies of the Mixteca Alta, Oaxaca, Mexico, using DNA sequences obtained from the partial gene *rbcLa*.

Sample ID	Morphological identification	*rbcLa*			
		Previous Record GeneBank	BLAST search best match	GeneBank accession number	Percent identity
AVM4	*Jamesonia flexuosa*	Yes	*Jamesonia flexuosa*	KJ416334.1	100.00
AVM30-01	*Lycopodium clavatum*	Yes	*Lycopodium clavatum*	KJ593516.1	100.00
			*Lycopodium japonicum*	MF786611.1	100.00
AVM31	*Diphasiastrum thyoides*	No	*Diphasiastrum digitatum*	MK525711.1	99.81
AVM42	*Marattia weinmanifolia*	Yes	*Marattia weinmanifolia*	EU221805.1	100.00
			*Marattia douglasii*	EU439083.1	100.00
AVM57	*Cyathea bicrenata*	Yes	*Cyathea valdecrenata*	AM410222.1	99.81
AVM68	*Diplazium lonchophyllum*	Yes	*Diplazium laffanianum*	KU363751.1	100.00
AVM77	*Dryopteris wallichiana*	Yes	*Dryopteris wallichiana*	KJ464428.1	99.81
AVM127	*Blechnum appendiculatum*	Yes	*Blechnum appendiculatum*	KU898613.1	100.00
AVM128	*Phanerophlebia macrosora*	No	*Phanerophlebia nobilis*	EF463214.1	98.77
AVM130	*Dryopteris wallichiana*	Yes	*Dryopteris wallichiana*	KJ464428.1	100.00
AVM132	*Asplenium monanthes*	Yes	*Asplenium monanthes*	AY300125.1	99.61
AVM154	*Lophosoria quadripinnata*	Yes	*Lophosoria quadripinnata*	KY684768.1	100.00
AVM155	*Marattia weinmanifolia*	Yes	*Marattia weinmanifolia*	EU221805.1	100.00
			*Marattia douglasii*	EU439083.1	100.00
AVM157	*Cyathea fulva*	Yes	*Cyathea fulva*	KY684773.1	100.00
			*Cyathea schiedeana*	AM410218.1	100.00
AVM171s	*Arachniodes denticulata*	yes	*Arachniodes denticulata*	KT272931.1	99.61
AVM224	*Cystopteris fragilis*	Yes	*Cystopteris fragilis*	JX874034.1	100.00
AVM228	*Polypodium conterminans*	No	*Polypodium plesiosorum*	FJ825696.1	100.00
AVM247	*Pteridium feei*	Yes	*Pteridium feei*	MK526462.1	100.00
AVM267g	*Gaga hirsuta*	No	*Gaga lerstenii*	JX313536.1	100.00
AVM268	*Polypodium subpetiolatum*	Yes	*Polypodium subpetiolatum*	KF909064.1	100.00
AVM277	*Pteris muricella*	No	*Pteris mertensioides*	KY099855.1	99.23
AVM280	*Asplenium monanthes*	Yes	*Asplenium monanthes*	AY300125.1	99.80
AVM288	*Elaphoglossum xanthopodum*	No	*Elaphoglossum lechlerianum*	EF463197.1	98.26
AVM303	*Adiantum andicola*	Yes	*Adiantum andicola*	KU147272.1	99.23
			*Adiantum feei*	MH019567.1	99.62
AVM304d	*Elaphoglossum petiolatum*	No	*Elaphoglossum huacsaro*	EF463195.1	99.60
AVM306	*Elaphoglossum xanthopodum*	No	*Elaphoglossum lechlerianum*	EF463197.1	98.26
AVM319	*Polystichum fournieri*	Yes	*Polystichum braunii*	MK526402.1	99.61
AVM403	*Blechnum appendiculatum*	Yes	*Blechnum appendiculatum*	KU898613.1	100.00
AVM404	*Cystopteris fragilis*	Yes	*Cystopteris fragilis*	JX874034.1	100.00

**Table 5:** BLAST search best match found on GeneBank for ferns and allies of the Mixteca Alta, Oaxaca, Mexico, using DNA sequences obtained from the intergenic spacer *trnH-psbA*.

Sample ID	Morphological identification	*trnH-psbA*			
		Previous record GeneBank	BLAST search best match	GeneBank accession number	Percent identity
AVM4	*Jamesonia flexuosa*	No	*Jamesonia brasiliensis*	MH173077.1	99.59
AVM30-01	*Lycopodium clavatum*	Yes	*Lycopodium clavatum*	NC_040994.1	99.30
AVM31	*Diphasiastrum thyoides*	Yes	*Diphasiastrum digitatum*	NC_040993.1	100.00
AVM42	*Marattia weinmanifolia*	Yes	*Marattia laxa*	NC_051979.1	99.04
AVM57	*Cyathea bicrenata*	No	*Cyathea epaleata*	KY099920.1	99.16
AVM68	*Diplazium lonchophyllum*	No	*Diplazium unilobum*	KY427347.1	95.24
AVM77	*Dryopteris wallichiana*	Yes	*Dryopteris goeringiana*	NC_050006.1	100.00
AVM127	*Blechnum appendiculatum*	No	*Blechnum occidentale*	MH178991.1	99.19
AVM128	*Phanerophlebia macrosora*	No	*Polystichum rigens*	AB575834.1	96.70
AVM130	*Dryopteris wallichiana*	Yes	*Dryopteris goeringiana*	NC_050006.1	100.00
AVM132	*Asplenium monanthes*	Yes	*Asplenium monanthes*	JQ767657.1	99.80
AVM154	*Lophosoria quadripinnata*	No	*Dicksonia antarctica*	JN575768.1	98.74
AVM155	*Marattia weinmanifolia*	No	*Marattia laxa*	NC_051979.1	97.73
AVM157	*Cyathea fulva*	No	*Cyathea epaleata*	KY099920.1	99.37
AVM171s	*Arachniodes denticulata*	Yes	*Arachniodes denticulata*	JN189425.1	99.77
AVM224	*Cystopteris fragilis*	Yes	*Cystopteris fragilis*	KU842451.1	97.36
AVM228	*Polypodium conterminans*	No	*Polypodium fauriei*	AB575897.1	97.91
AVM247	*Pteridium feei*	Yes	Pteridium aquilinum *	MF348630.1	100.00
AVM267g	*Gaga hirsuta*	No	*Gaga arizonica*	JN647842.1	98.59
AVM268	*Polypodium subpetiolatum*	No	*Polypodium fauriei*	AB575897.1	98.79
AVM277	*Pteris muricella*	No	*Pteris ensiformis*	AB575480.1	96.14
AVM279	*Polystichum fournieri*	Yes	*Polystichum paleatum*	KY099979.1	97.86
AVM280	*Asplenium monanthes*	Yes	*Asplenium monanthes*	JQ767629.1	100.00
AVM303	*Adiantum andicola*	No	*Adiantum shastense*	NC_037478.1	97.73
AVM304d	*Elaphoglossum petiolatum*	No	*Elaphoglossm samoense*	KY099932.1	98.36
AVM306	*Elaphoglossum xanthopodum*	No	*Elaphoglossm austromarquesense*	MT363025.1	95.48
AVM319	*Polystichum fournieri*	No	*Polystichum paleatum*	KY099979.1	97.86
AVM403	*Blechnum appendiculatum*	No	*Blechnum occidentale*	MH178991.1	98.99
AVM404	*Cystopteris fragilis*	Yes	*Cystopteris fragilis*	HQ157289.1	98.68

* *Pteridium aquilinum* is a basionym of *P. Feei*

A specimen data file, image file, and trace file(s) were submitted to BOLD along with edited and aligned sequences for each of our 29 samples of ferns and two samples of lycopods and can be accessed through the BOLD DNA database (http://www.boldsystems.org) under the ‘FERNO’ project. Twenty-nine fern sequences and two lycopod sequences were newly obtained in this study for *rbcLa* and *trnH-psbA* and BOLD ID numbers, and GeneBank accession numbers were generated (Table 6).

**Table 6:** Ferns and lycopods of the Mixteca Alta, Oaxaca, with their BOLD ID number and GeneBank accession number obtained from r*bcLa* and *trnH-psbA* amplifications, along with their sequence length.

Sample ID	Species	Process BOLD ID	*rbcLa *GeneBank accession number/ Sequence length bp	*trnH-psbA* GeneBank accession number/ Sequence length bp
AVM4	*Jamesonia flexuosa*	FERNO001-20	MZ771310 / 519	MZ870559 / 624
AVM30_01	*Lycopodium clavatum*	FERNO002-20	MZ771330 / 519	MZ870561 / 608
AVM31	*Diphasiastrum thyoides*	FERNO003-20	MZ771331 / 519	MZ870552 / 623
AVM42	*Marattia weinmanifolia*	FERNO004-20	MZ771329 / 519	MZ870563 / 623
AVM57	*Cyathea bicrenata*	FERNO005-20	MZ771314 / 519	MZ870548 / 623
AVM68	*Diplazium lonchophyllum*	FERNO006-20	MZ771324 / 519	MZ870553 / 604
AVM77	*Dryopteris wallichiana*	FERNO007-20	MZ771327 / 520	MZ870554 / 623
AVM127	*Blechnum appendiculatum*	FERNO008-20	MZ771318 / 519	MZ870546 / 623
AVM128	*Phanerophlebia macrosora*	FERNO009-20	MZ771335 / 488	MZ870564 / 623
AVM130	*Dryopteris wallichiana*	FERNO010-20	MZ771326 / 519	MZ870555 / 623
AVM132	*Asplenium monanthes*	FERNO011-20	MZ771307 / 519	MZ870545 / 623
AVM154	*Lophosoria quadripinnata*	FERNO012-20	MZ771332 / 519	MZ870560 / 623
AVM155	*Marattia weinmanifolia*	FERNO013-20	MZ771328 / 519	MZ870562 / 601
AVM157	*Cyathea fulva*	FERNO014-20	MZ771313 / 519	MZ870549 / 623
AVM171s	*Arachniodes denticulata*	FERNO015-20	MZ771333 / 519	MZ870543 / 623
AVM224	*Cystopteris fragilis*	FERNO016-20	MZ771320 / 519	MZ870551 / 608
AVM228	*Polypodium conterminans*	FERNO017-20	MZ771322 / 519	MZ870565 / 606
AVM247	*Pteridium feei*	FERNO018-20	MZ771317 / 519	MZ870569 /607
AVM267g	*Gaga hirsuta*	FERNO019-20	MZ771311 / 519	MZ870558 / 602
AVM268	*Polypodium subpetiolatum*	FERNO020-20	MZ771323 / 519	MZ870566 / 623
AVM277	*Pteris muricella*	FERNO021-20	MZ771312 / 519	MZ870570 / 649
AVM279	*Polystichum fournieri*	FERNO022-20	–	MZ870567 / 623
AVM280	*Asplenium monanthes*	FERNO023-20	MZ771308 / 519	MZ870544 / 623
AVM303	*Adiantum andicola*	FERNO024-20	MZ771309 / 520	MZ870542 / 607
AVM304d	*Elaphoglossum petiolatum*	FERNO025-20	MZ771334 / 500	MZ870556 / 600
AVM306	*Elaphoglossum xanthopodum*	FERNO026-20	MZ771316 / 519	MZ870557 / 600
AVM319	*Polystichum fournieri*	FERNO027-20	MZ771325 / 519	MZ870568 / 623
AVM403	*Blechnum appendiculatum*	FERNO028-20	MZ771319 / 519	MZ870547 / 623
AVM404	*Cystopteris fragilis*	FERNO029-20	MZ771321 / 519	MZ870550 / 607
AVM288	*Elaphoglossum xanthopodum*	FERNO030-20	MZ771315 / 519	–

### Genetic distance

**Figure 1:**
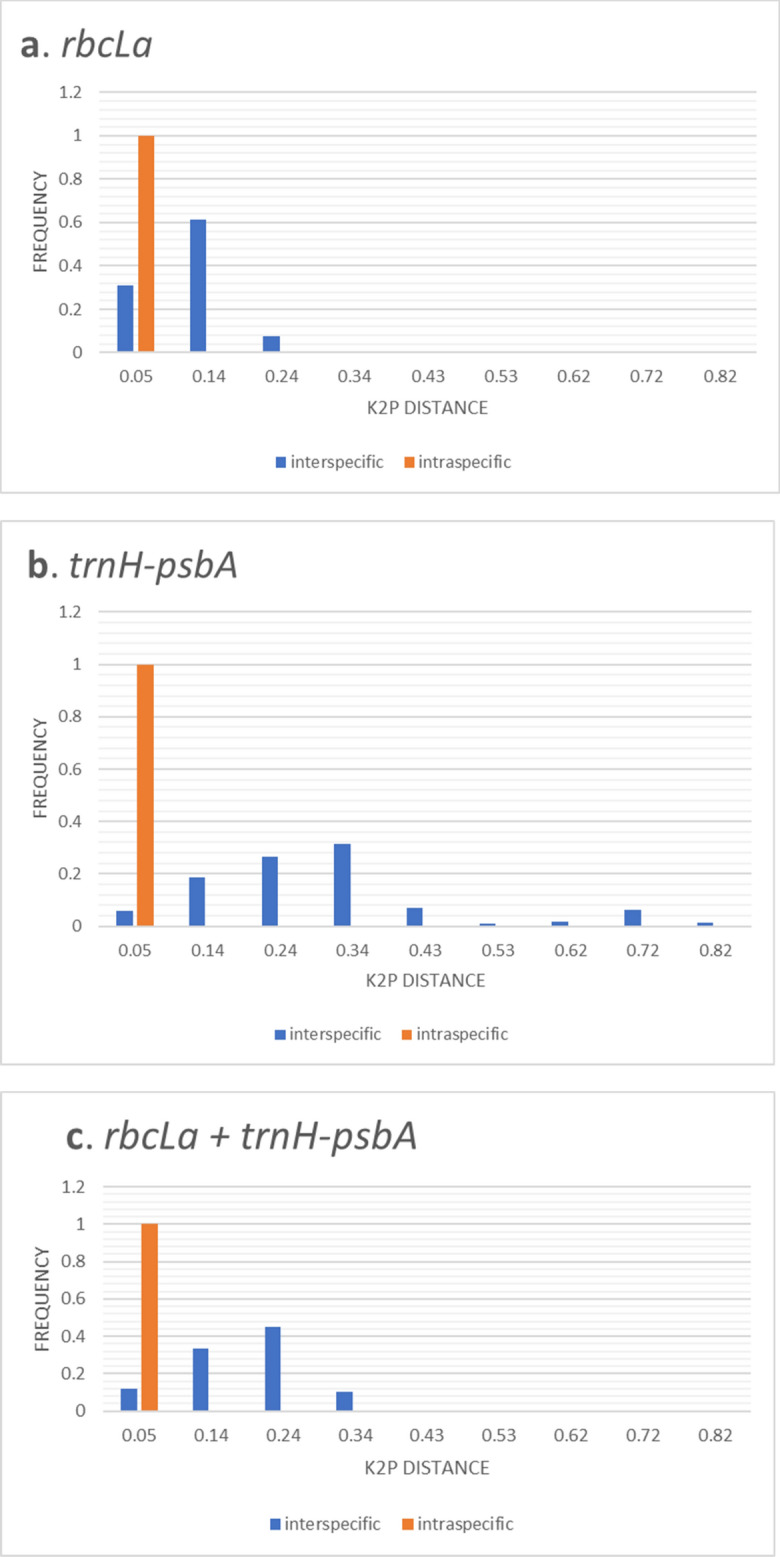
Distribution of interspecific and intraspecific K2P distances across all taxon pairs of ferns from Mixteca Alta, Oaxaca, obtained in partial gene ***rbcLa*** (**a**), intergenic spacer (**b**) and concatenated plastid regions (**c**).

The distribution of intraspecific and interspecific K2P distances across all taxon pairs of the ferns of The Mixteca Alta, Oaxaca, cloud forest, obtained from *rbcLa, trnH-psbA*, and combined DNA sequences of both plastid barcodes are shown in Fig. 1.

Based on previous work^33^, we included only one individual of each species to avoid biases created by an unequal number of sequences of each species. Intergenic spacer *trnH-psbA* had the highest mean interspecific K2P distance (0.3037 ± 0.1645 s.d.) in contrast to the mean values of *rbcLa* (0.1275 ± 0.0467 s.d.) and the combined DNA barcodes (0.1959 ± 0.0795 s.d.).

Results from the two-sample sign test in R of single and concatenated DNA sequences of *rbcLa* and *trnH-psbA* using tissues from different species of ferns and lycopods of the Mixteca Alta, Oaxaca, Mexico, are shown in Table 7. The intergenic spacer *trnH-psbA* showed the highest interspecific genetic divergence in comparison to *rbcLa* (median = − 0.1535, P value < 2.2e−16) and both plastid barcodes concatenated (median = 0.0851, P value < 2.2e−16).

**Table 7:** Two sample sign-test of interspecific divergence among loci and both plastid barcodes concatenated.

x	Y	Median x–y	95% confidence interval		n	P value	Result
*rbcLa*	*trnH-psbA*	− 0.1535	− 0.1662	− 0.1413	210	< 2.2e−16	*trnH-psbA* > *rbcLa*
*rbcLa*	*rbcLa* + *trnH-psbA*	− 0.0682	− 0.0728	− 0.0644	210	< 2.2e−16	*rbcLa* + *trnH-psbA* > *rbcLa*
*trnH-psbA*	*rbcLa* + *trnH-psbA*	0.0851	0.0772	0.0941	210	< 2.2e−16	*trnH-psbA* > *rbcLa* + trnH-psbA

### Topology results

**Figure 2:**
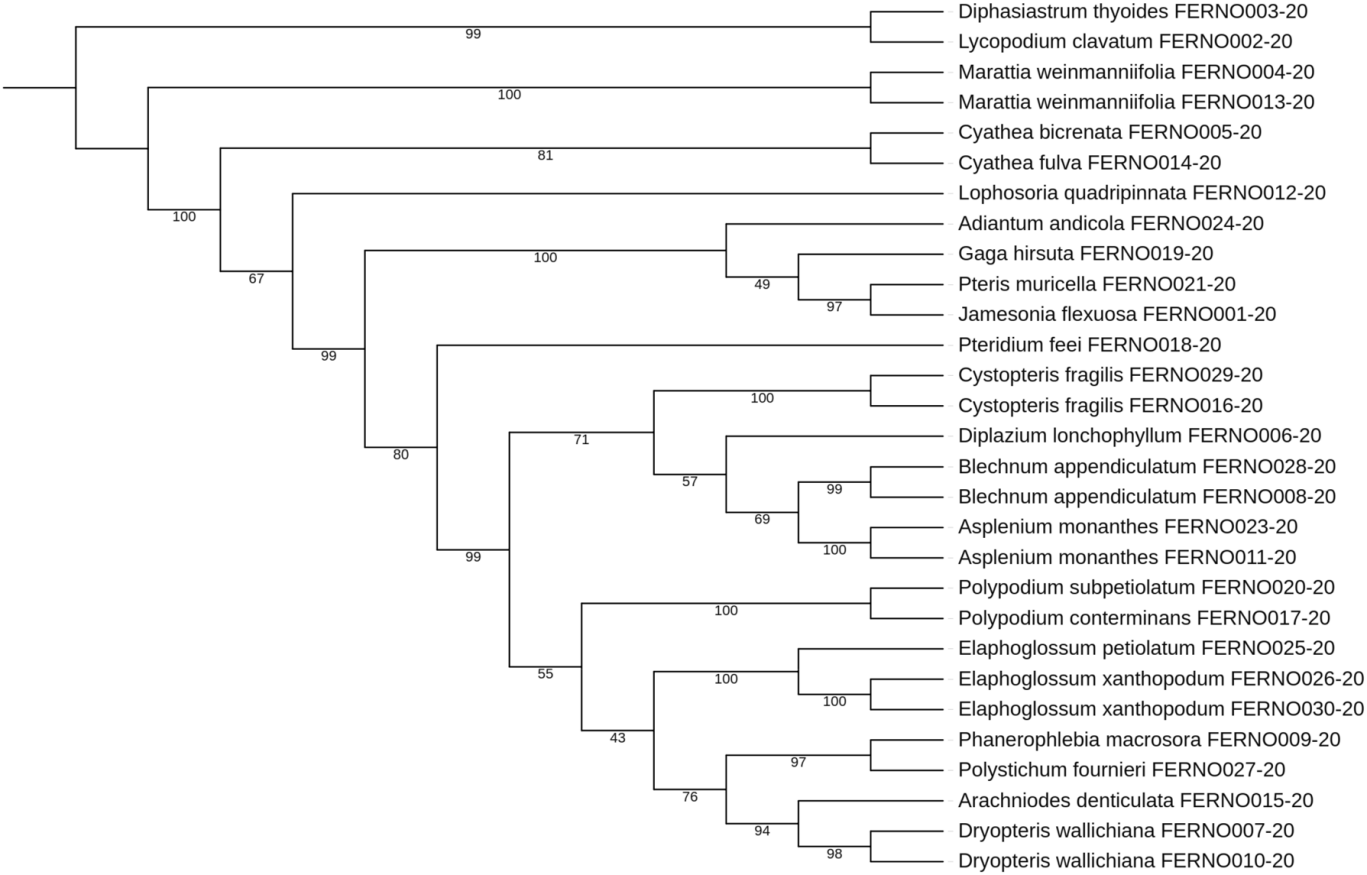
Maximum likelihood cladogram of plastid *rbcLa* for 27 sequences of ferns and 2 sequences of lycopods from Mixteca Alta, Oaxaca, México, tropical montane cloud forest. Bootstrap values based on 1000 replications are listed as percentages at branching points.

Phylogenetic tree-based analysis using neighbor-joining (Supplementary Fig. S1, Supplementary Fig. S2, Supplementary Fig. S3), maximum likelihood (Fig. 2, Supplementary Fig. S4, Supplementary Fig. S5), and Bayesian Inference trees (Supplementary Fig. S6, Supplementary Fig. S7, Supplementary Fig. S8) were reconstructed to evaluate ferns and lycopods species discrimination for the two barcode regions *rbcL* and *trnH-psbA*, single and combined (*rbcL* + *trnH-psbA*).

In the neighbor-joining trees, samples from *Polystichum fournieri* FERNO022-20 and *Elaphoglossum xanthopodum* FERNO030-20 were removed from the analysis of concatenated sequences since there were missing sequences in *rbcLa* data and *trnH-psbA*, respectively. The tree-based methods (NJ, ML, and BI) evaluated which tree produced the greatest species resolution and whether the barcode sequences generate monophyletic species (Table 8).

**Table 8:** Proportion (%) of monophyletic fern species and bootstrap or posterior probabilities, in parentheses, recovered with different phylogenetic techniques (NJ, ML, and BI) using single plastid barcodes *rbcLa* and *trnH-psbA* and combined DNA regions.

DNA region	NJ	ML	BI
*rbcLa*	100.00 (69.23)	100.00 (85.71)	82.61 (91.66)
*trnH-psbA*	100.00 (84.61)	100.00 (78.57)	34.78 (40.00)
*rbcLa* + *trnH-psbA*	100.00 (84.61)	100.00 (78.57)	82.61 (100.00)

NJ and ML phylogenetic trees resolved 100% of monophyletic species for *rbcLa*, *trnH-psbA,* and both barcodes combined (*rbcLa* + *trnH-psbA*) with a ≥ 70% clades support using bootstrap of 1000 replicates. The clade support value for *rbcLa* was higher in ML phylogenetic tree (85.71%) than in the NJ tree (69.23%), whereas the clade support value of *trnH-psbA* and *rbcLa* + *trnH-psbA* was higher in NJ trees (84.61%) than in the ML phylogenetic trees (78.57%). Since the mean clade support of all ML trees was 80.95%, and the mean clade support of all NJ trees was 79.49%, we conclude that the ML and NJ phylogenetic tree satisfactorily resolved the species monophyly of the studied ferns. We present the ML *rbcLa* phylogenetic tree (Fig. 2) since it yielded the most robust phylogeny: 85.71% of the nodes were supported by a maximum likelihood bootstrap ≥ of 70%.

All Bayesian Inference trees presented polytomies; *rbcLa* 1 (Supplementary Fig. S6), *trnH-psbA* 2 (Supplementary Fig. S7), and *rbcLa* + *trnH-psbA* 1 (Supplementary Fig. S8). With these polytomies, *rbcLa* could not resolve 4 monophyletic species, *trnH-psbA* 18 species, and *rbcLa* + *trnH-psbA* 4 species. Unlike the other two phylogenetic methods, BI using concatenated sequences showed an increase in clade support value.

## Discussion

Our amplification and sequencing results obtained with *rbcLa* and *trnH-psbA*, are very similar to those reported in other ferns studies^34–36^. Contrastingly, *matK* could not be amplified using two different sets of primers (Table 2). Although *matk* was proposed with *rbcL* as the barcode core for plants^5,37^, ferns appear to be the exception for this common finding. The failure of *matK* amplification in most leptosporangiate ferns using standard primers is most likely caused by a primer mismatch^8,12,20,35,38^. In most plants, *matK* is nested in the *trnK* intron, but *trnK* exons are lost in ferns^20,39^. For detailed studies, highly conserved exons in proximity with variable introns are convenient for phylogenetic analysis, allowing a high amplification efficiency of the primers situated in the exons and intron variability^40^. Due to the low primer universality of *matK* in ferns, many studies have designed different *matK* primers only for local ferns^12,20,41^. Because of the low amplification rates found in this and other studies^34,35^, we do not recommend the use of *matK* in ferns, except for particular situations.

Although we found a successful genera discrimination in these two plastid barcodes using BLASTn analysis, the low results for species discrimination are similar to those observed in ferns of Japan^42^, in which the rate of BLAST successful species discrimination for *rbcLa* and *trnH*-*psbA* was 70.91% and 65.05%, respectively. We could not find any improvement using both barcodes combined, which differs from results obtained in several studies of land plants^6,7,43^ and ferns^35,42^. Low rates of species identification using BLAST in our study are not necessarily caused by low marker performance. Four factors may contribute to explain these results. First, misidentified voucher specimens have been recognized as an increasing problem in public DNA databases, as several authors have acknowledged^10,28,44^. The rate of specimens correctly identified from the published samples is unknown. Second, online accessions in the GeneBank for our morphological species were limited. We could only find published sequences in 77% of the studied species for *rbcLa* and 33% for *trnH-psbA*. Indeed, new 27 *rbcLa* fern sequences and 27 *trnH-psbA* fern sequences along two lycopod sequences for each marker were submitted to BOLD along with its metadata.

Third, the widespread existence of hybridization and polyploidy in ferns^42,45,46^ is another factor that may decrease barcoding species discrimination^37^. Finally, translocation has been reported in some fern groups^41^. Other studies found a dramatically reduced *trnH-psbA* sequence variation for most ferns, probably due to the translocation of this segment into the plastid genome inverted repeat regions^41^. In our case, however, the intergenic spacer *trnH-psbA* displayed more interspecific K2P distances than those observed in *rbcLa* and the combined plastid barcodes (Fig. 1, Table 7). The faster rate of molecular divergence reported in several works^5,6,47^ for *trnH-psbA* than that for *rbcLa* in land plants may account for this result. Our results concur with those found in a recent meta-analysis using five major plant taxonomical groups^8^, which found a clear barcode gap on *trnH-psbA* sequences only in the fern group. Our two-sample sign test reveals that the intergenic spacer *trnH-psbA* offers better species discrimination than *rbcLa* and both plastid barcode combined for the studied group of ferns (Table 7).

We found similar results in barcode identification performance to those in other fern studies. For instance, higher interspecific variability in *trnH-psbA* than in *rbcLa* was also found in a study made in Moorea, French Polynesia with filmy ferns^36^, a work on Chinese medicinal pteridophytes^34^, and in studies involving several species of *Adiantum*^35^ and *Ophioglossum*^48^. However, some exceptions have been found. The mean interspecific divergence values across all taxon pairs (K2P genetic distances) in Japan’s pteridophytes^42^ did not reveal significant species discrimination between *trnH-psbA* and *rbcLa*. The *trnH-psbA* translocation mentioned above could partly explain these contrasting differences among different ferns studies reported only in certain groups of ferns.

From all topologies obtained in this work, maximum likelihood trees yielded the most robust phylogeny (Table 8). The phylogenetic arrangement found in our study concurs with a recent extant classification of ferns and lycopods^49^ and with other fern studies^42,50^. In all of our phylogenetic trees obtained for *rbcLa* and *trnH-psbA*, *Marattia weinmannifolia* is placed near the lycopods. The Marattiacea family is an eusporangiated and ancient group of ferns with fossil records extended back to the Middle Carboniferous^51^. In a recent study^52^, results of parsimony dating showed a minimum age estimate of 201–236 Ma, corresponding to late Triassic, for the most recent common ancestor of the extant Marattiaceae. Of all the ferns that we studied, the Marattiaceae is the most primitive, and this explains the higher similarity with the Lycopod outgroup, which is among the oldest groups of vascular plants^51^.

A paraphyletic clade was observed in the NJ *rbcLa* tree (Supplementary Fig. S1) and all three phylogenetic trees of *psbA-trnH* (Supplementary Fig. S2, Supplementary Fig. S4, Supplementary Fig. S7). *Elaphoglossum* (*E. xanthopodum* and *E. petiolatum*) was placed out of the Dryopteridacea family clade. The intergenic spacer *trnH-psbA* probably was more sensitive to nucleotide substitutions in this genus than *rbcLa*. A morphological and molecular study of the *Elaphoglossum* species^53^, which does not include our studied species, found that the relationship between *Elaphoglossum* with other fern genera is not clear. This genus was placed within Dryopteridaceae based on its chromosome number (x = 41) and monolete spores. However, in a recent extant fern classification based on new phylogenetic data^49^, *Elaphoglossum was* placed in a separate subfamily from the rest of the genera of Dryopteridaceae: Elaphoglossoideae. In agreement with such a decision, our phylogenetic trees using *trnH-psbA* could also successfully discriminate *Elaphoglossum* from other members of the Dryopteridaceae family.

## Conclusions

Based on the amplification capacity and sequence quality, the partial gene *rbcLa* and the intergeneric spacer *trnH-psbA* performed relatively well as barcode markers for ferns in the Mixteca Alta Oaxaca. Our ML phylogenetic trees agree with the recent extant lycophyte and fern phylogeny of the Pteridophyte Phylogeny Group (PPG). *rbcLa* outperforms in species discrimination and availability of sequences in public databases. However, *trnH-psbA* outperforms *rbcLa* in interspecific K2P distances and therefore could be helpful in some phylogenetic analysis involving groups without the inverted sequences translocation that may render low discrimination power. We did not find an increase in species discrimination using both plastid barcodes together in BLASTn, genetic distance, or any topology tree methods. Plastid barcode *matK* failed to successfully amplify fern and lycopod DNA sequences using universal primers. Our study pinpoints two problems: the low availability of DNA sequences for neotropical fern species and the need for more phylogenetic and polyploidy studies in ferns that clarify the phylogeny of certain groups, such as *Elaphoglossum*. We hope that the local barcode library that we generated could be the starting point for adding more sequences for a wide range of ecological, conservation, phylogenetic, and medical purposes.

## Supplementary Information

Supplementary Information.

## Data Availability

All generated ferns and lycopods DNA barcode sequences were registered at the GeneBank Taxonomy Database. Such sequences, the geographic coordinates of the specimens, and pictures of the plants were recorded in The Barcode of Life Data System (BOLD) (Table 6).
