# Supplementary material for: DNA barcoding ferns in an unexplored tropical montane cloud forest area of southeast Oaxaca, Mexico
Source: Sci Rep. 2021 Nov 24;11:22837. doi: 10.1038/s41598-021-02237-8 (PMC8613246; doi:10.1038/s41598-021-02237-8)
Supplement: Supplementary file 1 — Supplementary Information. [file 41598_2021_2237_MOESM1_ESM.pdf]

DNA barcoding ferns in an unexplored tropical montane cloud forest area of southeast Oaxaca, Mexico.

Sonia Trujillo-Argueta, Rafael F. del Castillo, Daniel Tejero-Diez, Carlos Alberto Matias-Cervantes and Abril Velasco-Murguía.

Supplementary Figures

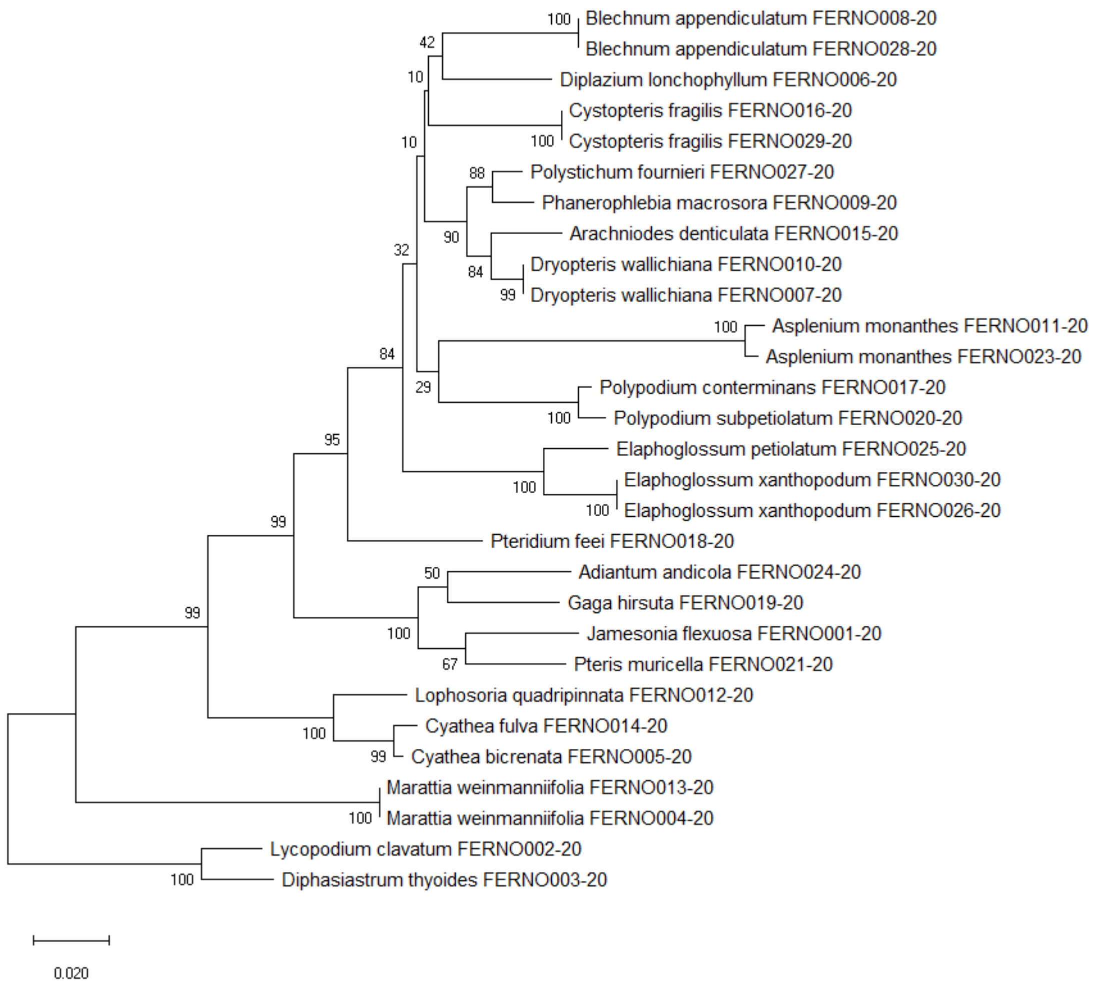

Supplementary Figure S1. Neighbor-Joining phylogram for *rbcLa* for 27 ferns sequences and 2 lycopods from Mixteca Alta, Oaxaca, México. The percentage of replicate trees in which the associated taxa clustered together in the bootstrap test (1000 replicates) are shown next to the branches.

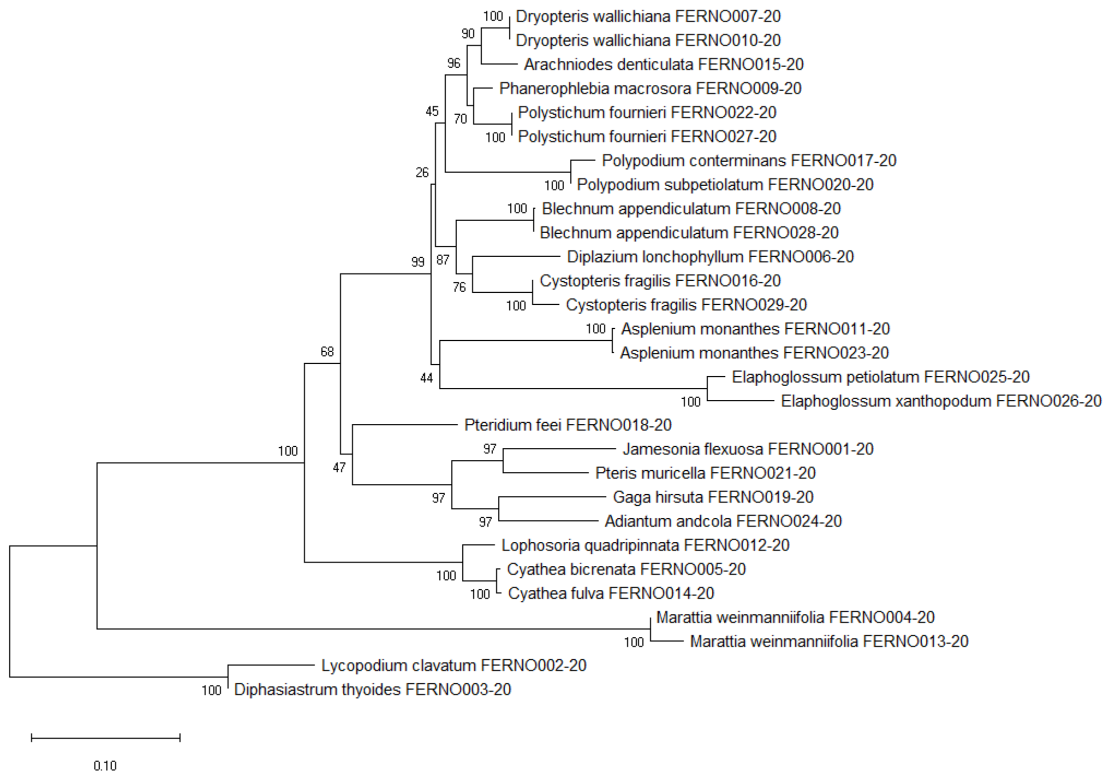

Supplementary Figure S2. Neighbor-Joining phylogram for *trnH-psbA* for 27 ferns sequences and 2 lycopods from Mixteca Alta, Oaxaca, México. The percentage of replicate trees in which the associated taxa clustered together in the bootstrap test (1000 replicates) are shown next to the branches.

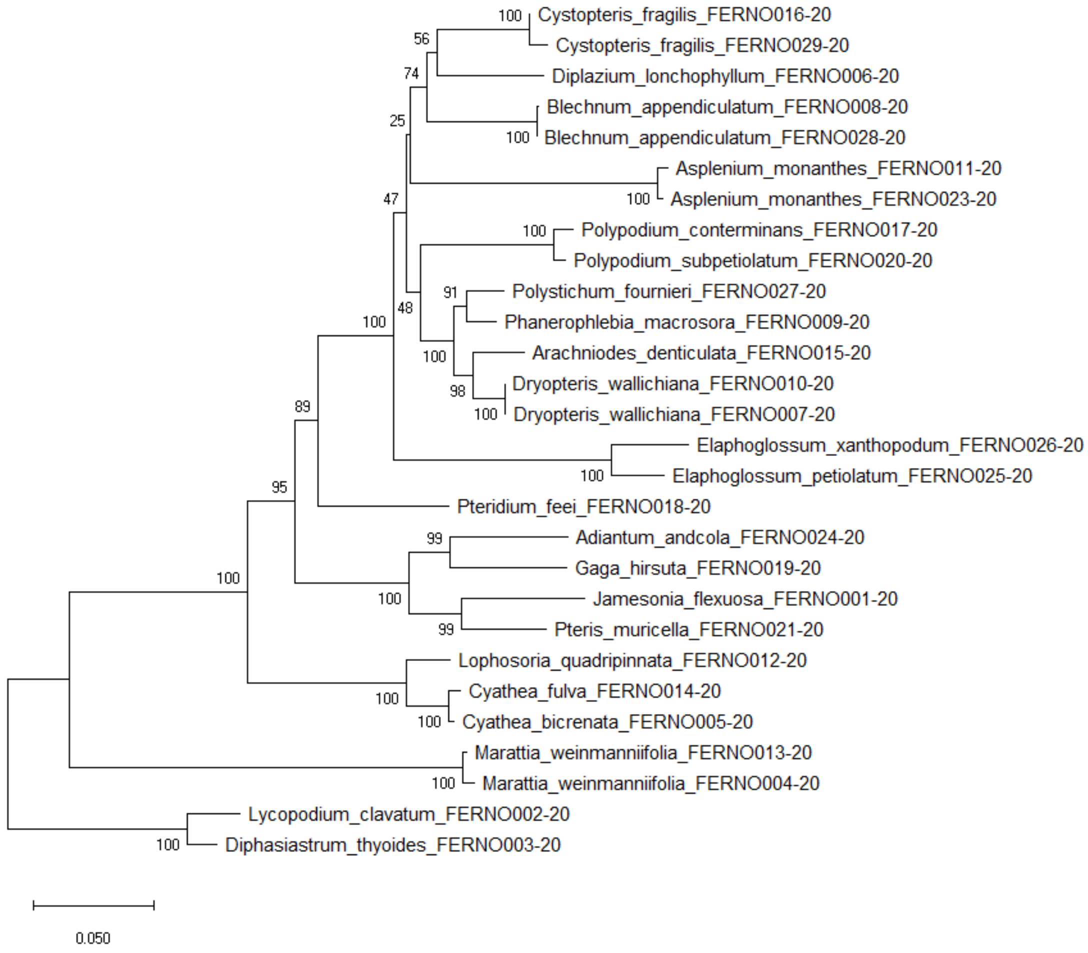

Supplementary Figure S3. Neighbor-Joining phylogram for concatenated sequences *rbcLa* + *trnH-psbA* for 26 ferns sequences and 2 lycopods from Mixteca Alta, Oaxaca, México. The percentage of replicate trees in which the associated taxa clustered together in the bootstrap test (1000 replicates) are shown next to the branches.

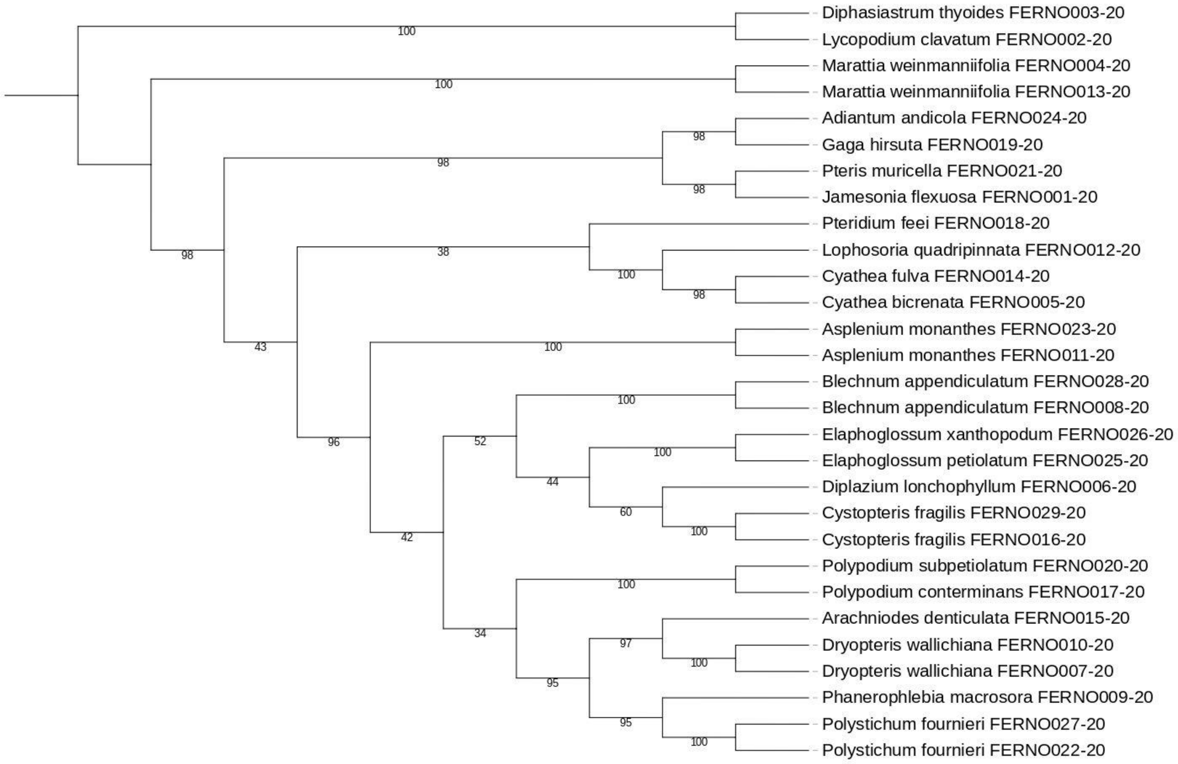

Supplementary Figure S4. Maximum likelihood cladogram of plastid *trnH-psbA* for 27 sequences of ferns and 2 sequences of lycopods from Mixteca Alta, Oaxaca, México. Bootstrap values based on 1000 replications are listed as percentages at branching points.

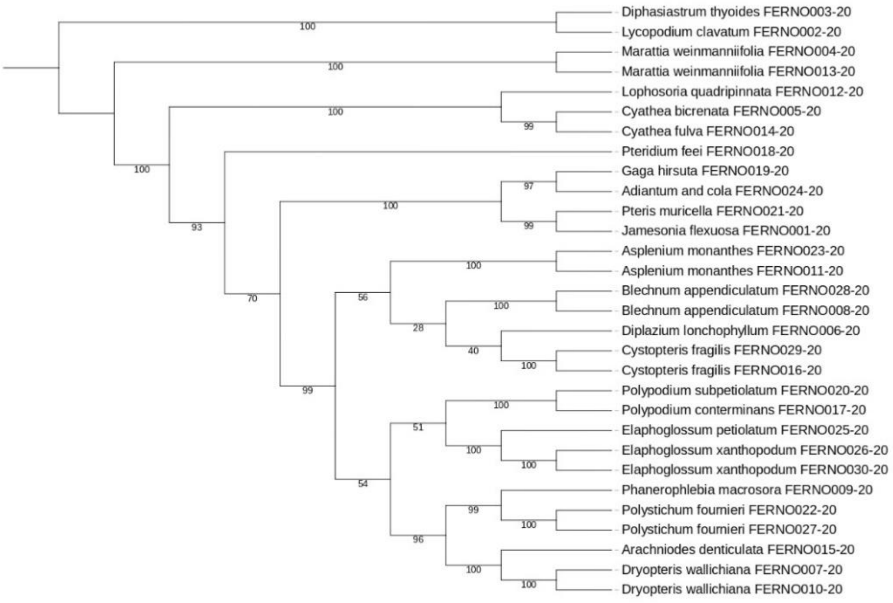

Supplementary Figure S5. Maximum likelihood cladogram of plastid barcodes *rbcL* + *trnH-psbA* for 28 sequences of ferns and 2 sequences of lycopods from Mixteca Alta, Oaxaca, México. Bootstrap values based on 1000 replications are listed as percentages at branching points.

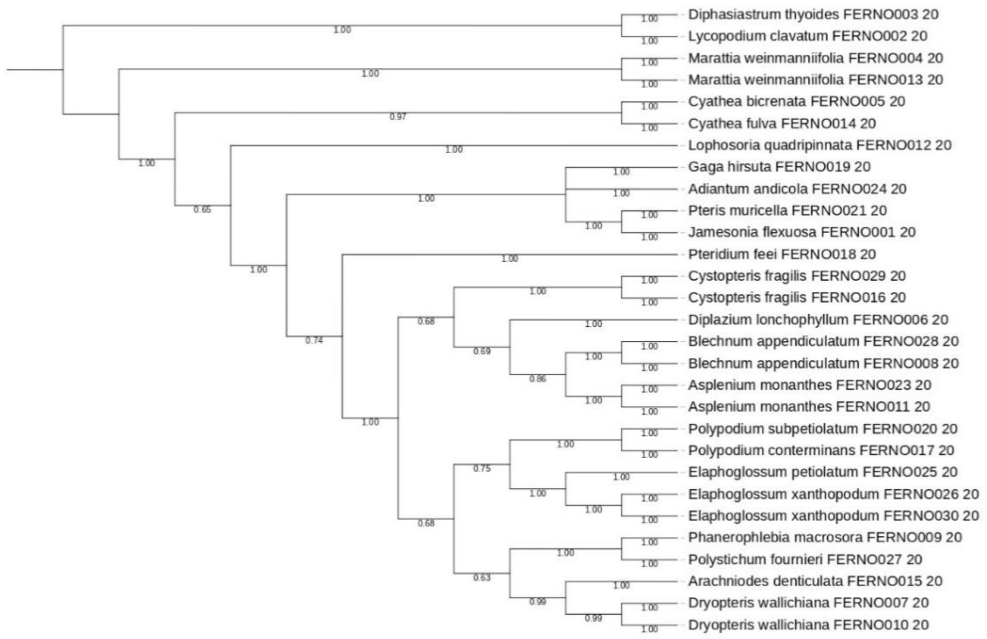

Supplementary Figure S6. Bayesian inference tree for *rbcLa* for 27 ferns sequences and 2 lycopods sequences from Mixteca Alta, Oaxaca, México. Bayesian posterior probabilities are below the branches.

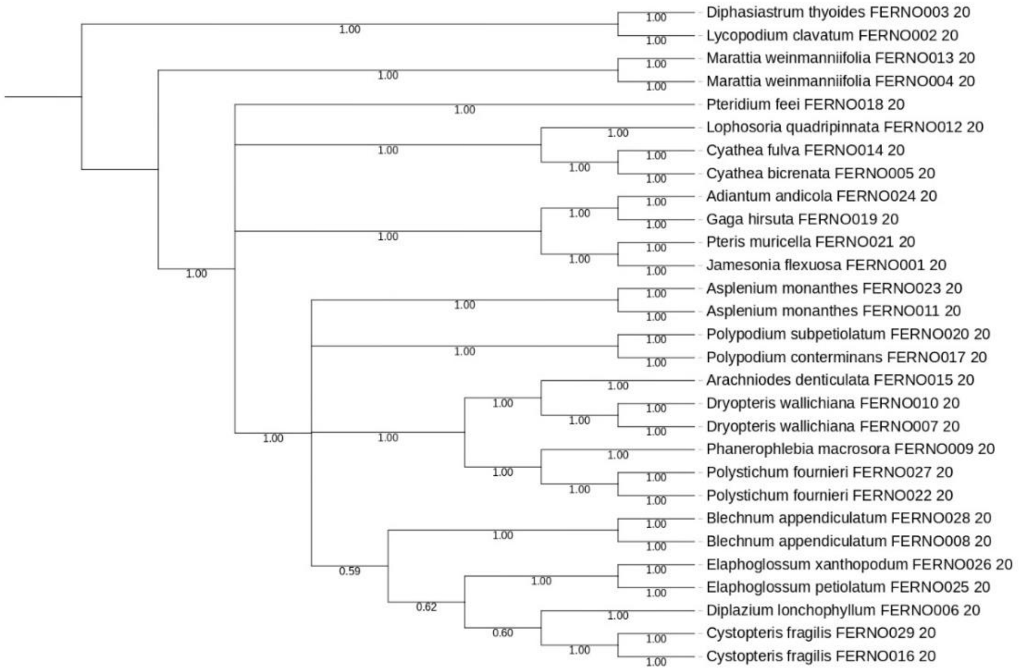

Supplementary Figure S7. Bayesian inference tree for *trnH-psbA* for 27 ferns sequences and 2 lycopods sequences from Mixteca Alta, Oaxaca, México. Bayesian posterior probabilities are below the branches.

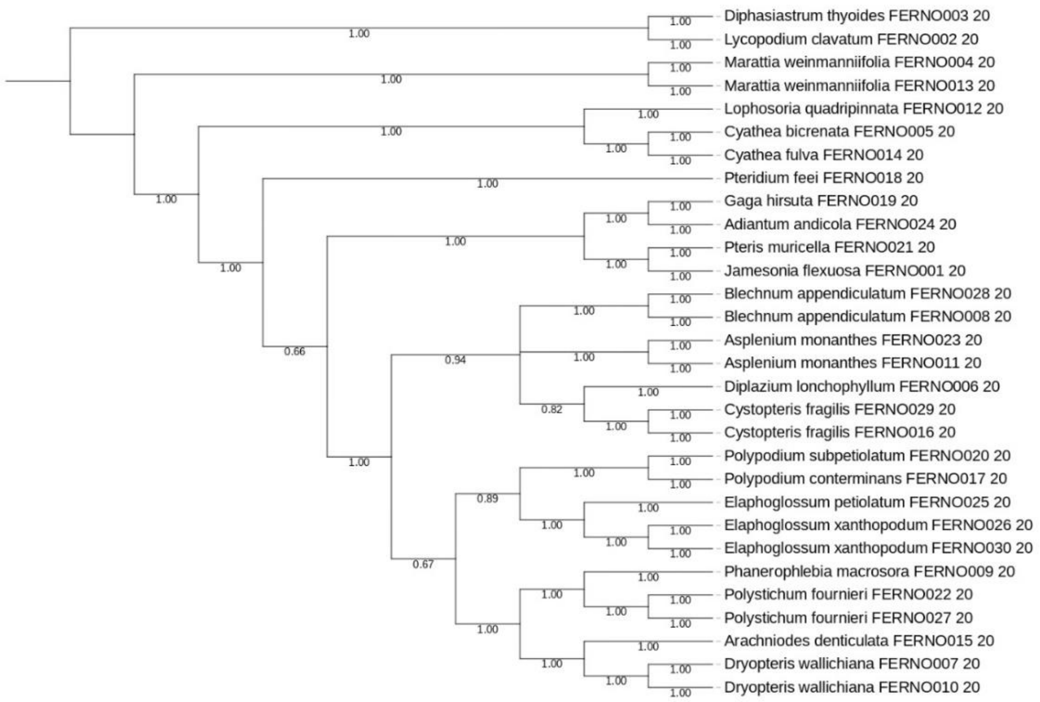

Supplementary Figure S8. Bayesian inference tree based on concatenated *rbcLa* + *trnH-psbA* of 28 ferns sequences and 2 lycopods sequences from Mixteca Alta, Oaxaca, México. Bayesian posterior probabilities are below the branches.
